# The factors affecting the evolution of the anthocyanin biosynthesis pathway genes in monocot and dicot plant species

**DOI:** 10.1186/s12870-017-1190-4

**Published:** 2017-12-28

**Authors:** Olesya Yu. Shoeva, Anastasiya Yu. Glagoleva, Elena K. Khlestkina

**Affiliations:** 1 Institute of Cytology and Genetics, Siberian Branch, Russian Academy of Sciences, Novosibirsk, Russia; 2 Novosibirsk State University, Novosibirsk, Russia

**Keywords:** Anthocyanin biosynthesis, Dicots, Monocots, Gene regulation, Pollination type, Selective constraint

## Abstract

**Background:**

The available data demonstrate that even in universal metabolic pathways, some species-specific regulatory features of structural genes are present. For instance, in the anthocyanin biosynthesis pathway (ABP), genes may be regulated by ABP-specific regulatory factors, and their expression levels may be strongly associated with anthocyanin pigmentation, or they may be expressed independently of pigmentation. A dataset of orthologous ABP genes (*Chs*, *Chi*, *F3h*, *F3’h*, *Dfr*, *Ans*) from monocot and dicot plant species that have distinct gene regulation patterns and different types of pollination was constructed to test whether these factors affect the evolution of the genes.

**Results:**

Using a maximum likelihood approach, we demonstrated that although the whole set of the ABP genes is under purifying selection, with greater selection acting on the upstream genes than on the downstream genes, genes from distinct groups of plant species experienced different strengths of selective pressure. The selective pressure on the genes was higher in dicots than in monocots (*F3h* and further downstream genes) and in pollinator-dependent plants than in pollinator-independent species (*Chi* and further downstream genes), suggesting an important role of pollination type in the evolution of the anthocyanin biosynthesis gene network. Contrasting effects of the regulation patterns on evolution were detected for the *F3h* and *Dfr* genes, with greater selective pressure on the *F3h* gene in plant species where the gene expression was not strongly associated with pigmentation and greater selective pressure on *Dfr* in plant species where the gene expression was associated with pigmentation.

**Conclusions:**

We demonstrated the effects of pollination type and patterns of regulation on the evolution of the ABP genes, but the evolution of some of the genes could not be explained in the framework of these factors, such as the weaker selective pressure acting on *Chs* in species that attract pollinators or the stronger selective pressure on *F3h* in plant species where the gene expression was not associated with pigmentation. The observations suggest that additional factors could affect the evolution of these genes. One such factor could be an effect of gene duplication with further division of functions among gene copies and relaxed selective pressure acting on them. Additional tests with an appropriate dataset combining data on duplicated gene sequences and their functions in the flavonoid biosynthesis pathway are required to test this hypothesis.

**Electronic supplementary material:**

The online version of this article (10.1186/s12870-017-1190-4) contains supplementary material, which is available to authorized users.

## Background

The anthocyanin biosynthesis pathway (ABP) produces the pigments responsible for the coloration of different parts of a plant. It shares a number of common enzymes with pathways that produce diverse classes of flavonoids (Additional file 1: Figure S1), which serve important physiological functions, including protection from pathogens and unfavorable environmental factors, signaling and interactions with symbionts, promotion of pollen tube growth, and mediation of hormone transport [1–3]. The structural genes of the ABP are common to all angiosperms and have been characterized in many plant species. They can be divided into two groups: upstream genes, encoding chalcone synthase (CHS), chalcone-flavanone isomerase (CHI), flavanone 3-hydroxylase (F3H), and dihydroflavonol 4-reductase (DFR), synthesize precursors for one or more non-anthocyanin flavonoid pathways, and downstream genes, encoding anthocyanidin synthase (ANS), glycosyltransferase (GT), rhamnosyltransferase (RT), acetyltransferase (AT), and methyltransferase (MT), are specific to the anthocyanin pathway (Additional file 1: Figure S1).

The ABP is temporally and spatially regulated by transcription factors belonging to three main groups of regulatory factors (MYB, MYC (bHLH), and WD40) that generate the MBW complex required for activating structural genes [4].

As one of the best-characterized metabolic pathways, the ABP gene network provides a useful system for studying the patterns of the evolution of genes within the same metabolic pathway [5]. The slower evolution of upstream enzymes compared with downstream ones have been demonstrated across a broad taxonomic distance involving comparisons among monocots and dicots as well as in the genus *Ipomoea*, specifically [6, 7]. One of the possible explanations for this evolutionary pattern is that the upstream enzymes are under greater selective constraint (i.e., under greater strength of purifying selection) than the downstream enzymes. The greater constraint may arise because the upstream genes participate in the synthesis of diverse classes of flavonoids. Mutations in these genes would have more deleterious pleiotropic effects on plant fitness and would be more likely to be eliminated by purifying selections, unlike mutations occurring in downstream genes, which could persist in a population [6–8]. Similar patterns of progressive relaxation of selective constraints along metabolic pathways have been reported for terpenoid [9] and carotenoid [10] biosynthesis pathways. However, no relationships between the position of the genes in a pathway and selective constraints have been found when studying the phenylpropanoid pathway in *Arabidopsis thaliana* [11], the gibberellin pathway in the Oryzeae tribe [12], or the starch pathway in *Oryza sativa* [13].

Another evolutionary pattern is more rapid evolution of the regulatory genes of the ABP compared with the structural genes [6]. Although no evidence of positive selection in the MYB, MYC (bHLH), and WD40 regulatory genes have been obtained, the data have demonstrated variation in selective constraints on the regulatory genes, with more relaxed (weaker) selection on MYB genes. The difference in selective constraints experienced by regulatory genes has also been explained by their different functional loads, with greater selective pressure acting on genes having more functions [14].

Another evolutionary pattern of the ABP genetic network has been revealed by Ho and Smith [15], who studied the effects of floral pigmentation losses in the evolution of the structural genes of the pathway. They found that although the genes from both pigmented and unpigmented lineages of Solanaceae experienced purifying selection, relaxed selective pressures on two of the three structural genes studied (*Chi* and *F3h*) were detected in unpigmented lineages in comparison with the genes from the pigmented lineages. Assuming the pigmentation losses are due to mutations in regulatory genes, we hypothesized that there is a link between the evolution of structural and regulatory genes of the ABP.

**Fig. 1:**
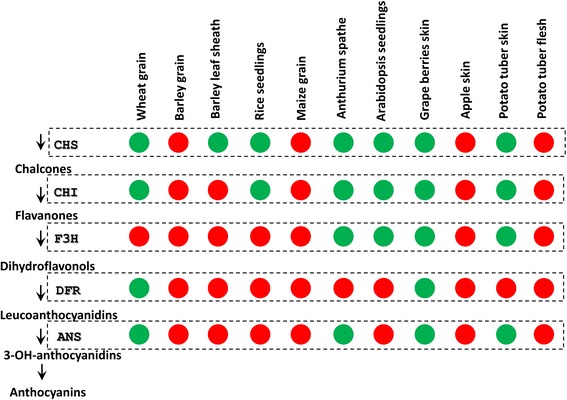
Regulation patterns of the anthocyanin biosynthesis pathway genes in different plant species. The red circles show the regulation of the genes dependent on anthocyanin biosynthesis-specific transcription factors and pigment accumulation; the green circles mark the genes expressed independently of pigmentation

The available data demonstrate that in universal anthocyanin biosynthesis metabolic pathways, some species-specific regulatory features of the structural genes occur. The common structural genes are regulated differently in various plant species, depending on the expression of regulatory genes that predetermine pigmentation in different organs (Fig. 1). In some plant species, for example, maize (*Zea mays*) and barley (*Hordeum vulgare*), the whole set of ABP genes is co-regulated by the MBW complex as one regulatory unit [16, 17]. In other plant species, just a portion of these genes or even a single gene is strongly regulated by the ABP regulatory factors, whereas the genes in the rest of the pathway have distinct patterns of regulation independent of the ABP regulatory factors and anthocyanin pigmentation. Such patterns have been described, for example, in *Arabidopsis thaliana* seedlings, where the genes are divided into genes of ‘early’ and ‘late’ biosynthesis. The ‘early’ genes are expressed in the absence of pigment synthesis, while the ‘late’ genes are co-expressed (jointly regulated) in the presence of the anthocyanin biosynthesis-specific MBW complex [18].

We hypothesized that the regulation pattern that determines development of pigmentation could create additional selective constraints on genes involved in pigmentation in comparison with genes that are regulated independently of pigmentation. The current study was conducted to test whether the different regulation modes affect the molecular evolution of the ABP structural genes. The dataset was constructed based on monocotyledonous and dicotyledonous plant species with the most characterized structural genes and their regulatory patterns. The dataset allows testing of additional factors that could affect molecular evolution. Particularly, the selective constraint on the ABP genes were tested to determine differences between monocot and dicot plant species classes and how pollination type affects ABP gene evolution.

## Results

### Identification of the orthologous set of anthocyanin biosynthesis genes and their regulatory patterns

Sequences of the six ABP genes (*Chs*, *Chi*, *F3h*, *F3’h*, *Dfr*, *Ans*) in monocots (*Aegilops tauschii* Coss., *Anthurium andraeanum* Linden ex André, *Hordeum vulgare* L., *Oryza sativa* L., *Triticum aestivum* L., *Triticum urartu* Thum. ex Gandil., *Zea mays* L.) and dicots (*Arabidopsis lyrata* (L.) O’Kane & Al-Shehbaz, *Arabidopsis thaliana* (L.) Heynh., *Malus domestica* Borkh., *Solanum tuberosum* L., *Vitis amurensis* Rupr., *Vitis vinifera* L.), with the most characterized pathways of structural genes, and their regulatory patterns were retrieved from databases (Additional file 2: Table S1). The lengths of the coding regions of the chosen plant species varied from 1170 to 1203 bp in *Chs*, from 687 to 777 bp in *Chi*, from 1077 to 1167 bp in *F3h*, from 1530 to 1671 bp in *F3’h*, from 1014 to 1155 bp in *Dfr*, and from 1065 to 1368 bp in *Ans*. The variation in length between the genes was predetermined by insertion-deletion (indel) events that occurred during evolution. These indels were excluded from the analysis.

**Fig. 2:**
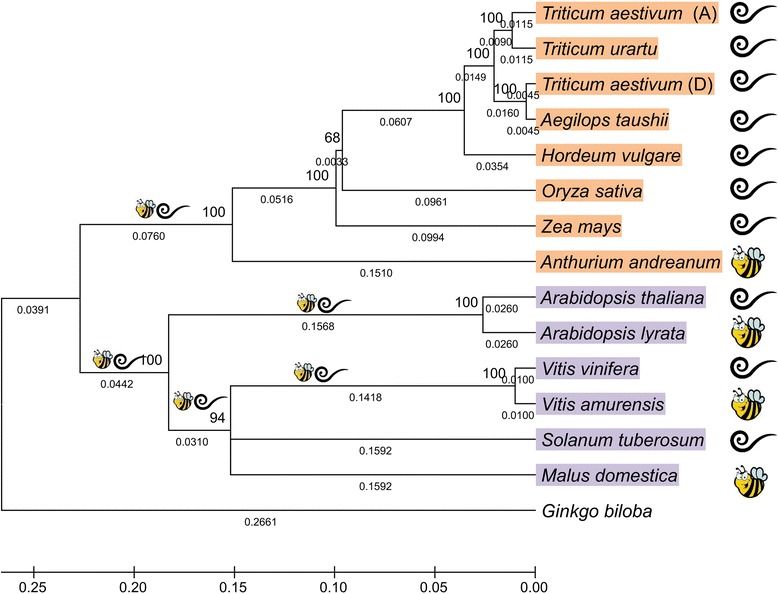
Phylogeny of members of dicotyledonous and monocotyledonous plant species chosen for the analysis. The scale and the branch lengths represent the number of nucleotide substitutions per site. The numbers at the nodes are bootstrap values performed with 1000 replications. The combination of the species based on the pollination type is shown: bees mark species pollinated predominantly by pollinators, spirals mark species pollinated without attracting pollinators

Across all genes, the pairwise divergence at the nucleotide level within monocot and dicot plant species classes varied from 0 to 45% (in *Chi*). Between the monocots and dicots, the divergence reached up to 65% (in *Ans*) (Additional file 3: Figure S2). For molecular evolution analysis, a phylogenetic tree was constructed based on combined ABP genes for each species (Fig. 2). The groups of monocots and dicots were distinguished in the tree, and the tree corresponded to the taxonomic classifications of the plant species chosen for the analysis.

Among the plant species selected for the analysis, the patterns of ABP gene regulation were distinct (Fig. 1). The co-regulation of the whole set of genes by ABP regulatory factors has been reported to occur in grains of maize and barley [16, 17], in the skin of apples (*M. domestica*) [19] and in the flesh of potato (*S. tuberosum*) tubers [20]. Two types of differentially regulated genes, distinguished by their dependence on the co-regulation with the ABP regulatory factors or their expression independent of the regulatory factors, have been revealed in barley leaf sheaths [21], seedlings of rice (*O. sativa*) [22] and *Arabidopsis* [18], wheat grains [23], skins of potato tubers [24, 25], spathes of flamingo flowers (*A. andraeanum*) [26], and skins of grape berries [27, 28]. In conclusion, the regulation mode of the ABP genes is species specific and is not dependent on the class of the plant species.

### Variation in selective pressures on the ABP genes

A single-ratio model of non-synonymous to synonymous substitution rates (dN/dS or ω) for each gene showed that the ω values varied from 0.036 (for *Chs*) to 0.121 (*Chi*) (Table 1), suggesting that these genes predominantly experienced purifying selection. The dN/dS ratios were compared between genes (Additional file 4: Table S2). *Chs* experienced the strongest selective pressure (dN/dS = 0.036), which was significantly lower than the selective pressures acting on the other ABP genes. Less constrained selection was acting on *F3h* (0.061), which significantly differed from the other genes. The group of the genes *F3’h*, *Dfr* and *Ans* demonstrated significantly indistinguishable selective pressures varying from 0.085 to 0.095, which differed significantly from the other genes. The weakest selective pressure was detected for the *Chi* gene (0.121). There was no correlation between the dN/dS ratio of the structural genes and positions of the enzymes encoded by the genes in the ABP metabolic pathway (Table 2, global).

**Table 1:** Maximum likelihood tests of selection on *Chs*, *Chi*, *F3h*, *F3’h*, *Dfr*, and *Ans* using HyPhy

Gene	Model	№ of parameters	Ln *L*	ω
*Chs*	Global	33	−7585.83	ω = 0.036
	Local	59	−7532.90**	
	Class (monocots *vs* dicots)	35	−7583.59	ω_m_ = 0.035, ω_d_ = 0.039
	Pollination (pollinators *vs* no-pollinators)	36	−7577.00**	ω_p_ = 0.099, ω_np_ = 0.042
	Regulation (color-dep *vs* color-indep)	35	−7582.90	ω_cd_ = 0.051, ω_cid_ = 0.035
*Chi*	Global	33	−4367.90	ω = 0.121
	Local	59	−4349.01**	
	Class (monocots *vs* dicots)	35	−4366.33	ω_m_ = 0.163, ω_d_ = 0.104
	Pollination (pollinators *vs* no-pollinators)	36	−4362.81*	ω_p_ = 0.102, ω_np_ = 0.173
	Regulation (color-dep *vs* color-indep)	35	−4367.80	ω_cd_ = 0.136, ω_cid_ = 0.119
*F3h*	Global	33	−5750.13	ω = 0.061
	Local	59	−5685.06**	
	Class (monocots *vs* dicots)	35	−5731.97**	ω_m_ = 0.125, ω_d_ = 0.034
	Pollination (pollinators *vs* no-pollinators)	36	−5717.46**	ω_p_ = 0.058, ω_np_ = 0.117
	Regulation (color-dep *vs* color-indep)	35	−5723.24**	ω_cd_ = 0.127, ω_cid_ = 0.032
*F3’h*	Global	33	−10,269.21	ω = 0.085
	Local	59	−10,230.12**	
	Class (monocots *vs* dicots)	35	−10,257.69**	ω_m_ = 0.120, ω_d_ = 0.065
	Pollination (pollinators *vs* no-pollinators)	36	−10,259.64**	ω_p_ = 0.068, ω_np_ = 0.112
	Regulation (color-dep *vs* color-indep)	No data		
*Dfr*	Global	33	−6728.67	ω = 0.095
	Local	59	−6661.18**	
	Class (monocots *vs* dicots)	35	−6701.70**	ω_m_ = 0.201, ω_d_ = 0.061
	Pollination (pollinators *vs* no-pollinators)	36	−6703.59**	ω_p_ = 0.114, ω_np_ = 0.161
	Regulation (color-dep *vs *color-indep)	35	−6722.73*	ω_cd_ = 0.098, ω_cid_ = 0.107
*Ans*	Global	33	−7629.37	ω = 0.094
	Local	59	−7547.03**	
	Class (monocots *vs* dicots)	35	−7588.21**	ω_m_ = 0.219, ω_d_ = 0.049
	Pollination (pollinators *vs* no-pollinators)	36	−7585.99**	ω_p_ = 0.067, ω_np_ = 0.196
	Regulation (color-dep *vs* color-indep)	35	−7626.46	ω_cd_ = 0.087, ω_cid_ = 0.106

Three factors were tested: plant classes (two separate ω were calculated for monocotyledonous and dicotyledonous plant species, ω_m_/ω_d_); pollination type (the ω for the plant species pollinated by pollinators was calculated separately from the plant species pollinated without attracting pollinators, ω_p_/ω_np_); regulation pattern (the plant species were combined based on color-dependent and color-independent ABP gene regulation, ω_cd/_ω_ind_). Significant improvement in the likelihood in comparison with the one-ratio model (Global) is indicated with asterisks: **p < 0.001, *p < 0.05

**Table 2:** Spearman’s rank correlation coefficients between the dN/dS ratios of the genes and the positions of the enzymes encoded by the genes in the ABP metabolic pathway in plant species combined based on the taxonomy, pollination type or regulation mode

	Groups of species	With *Chi*	Without *Chi*
	global	0.36	0.87
Taxonomy	monocots	0.81*	0.97*
	dicots	−0.03	0.11
Pollination	no pollinators	0.64	0.97*
	pollinators	−0.20	−0.10
Regulation	color-dependent	ND	0.20
	color-independent	0.10	0.60

*Significant at *p* < 0.05

### The factors affecting the selective pressures on the ABP genes

The plant species were combined into two groups based on whether they belonged to the monocot or dicot class (Fig. 2), pollination type (Fig. 2), or regulation mode (Fig. 1). Branch models with separate dN/dS ratios for these groups were applied in order to test the hypothesis that these factors could have an impact on the selective pressures acting on the genes.

**Fig. 3:**
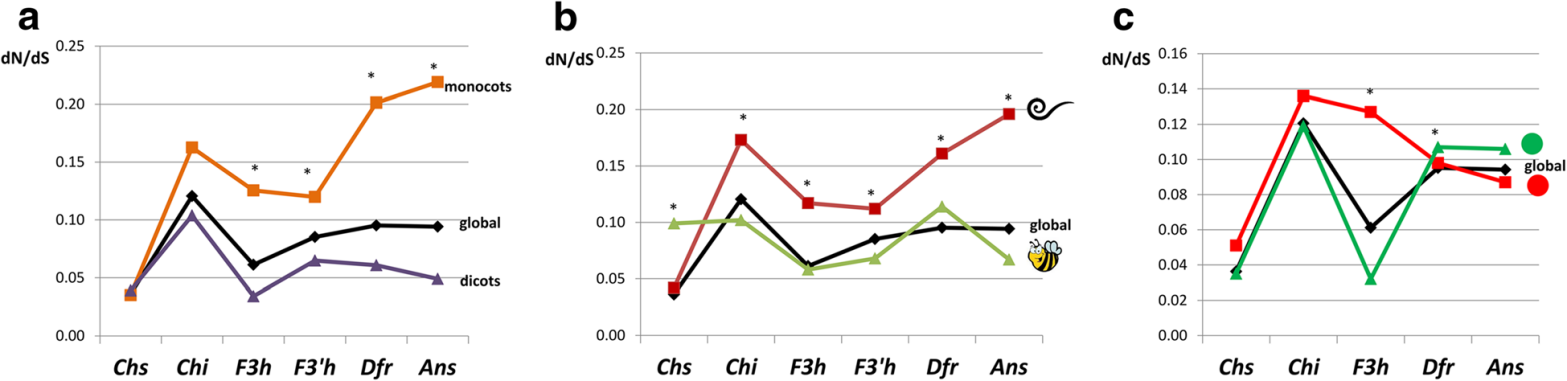
Patterns of variation in the dN/dS ratio across genes and between groups of the plant species combined based on the plant classes (**a**), pollination type (**b**) and regulation mode (**c**). ‘Global’ shows dN/dS values for the single ratio model for each of the genes

To distinguish between monocots and dicots, the two-ratio models were used and revealed significantly higher dN/dS ratios in monocots compared to dicots for *F3h* (0.125 *vs* 0.034), *F3’h* (0.120 *vs* 0.065), *Dfr* (0.201 *vs* 0.061), and *Ans* (0.219 *vs* 0.049). Two-ratio models for these genes resulted in significantly higher likelihoods than the single (global) ratio models, whereas the likelihood function was not improved under the two-ratio models for *Chs* and *Chi* (Table 1, Fig. 3a); hence, the single ratio model could not be rejected. The strong positive correlation between the dN/dS ratios of the structural genes and positions of the enzymes encoded by the genes in the metabolic pathway was detected in the monocotyledonous plant species group (*r*_*s*_ = 0.81, *p* < 0.05), whereas in the dicotyledonous group, the correlation was absent (*r*_*s*_ = −0.03, *p* > 0.05) (Table 2).

Pollination type was the second factor tested that could affect the selective pressures acting on the ABP genes. The three-ratio models revealed significantly higher dN/dS ratios for the genes in plant species pollinated without pollinators compared to the species pollinated by attracting pollinators (Table 1, Fig. 3b), except for *Chs*, which had a dN/dS ratio in the group pollinated by pollinators that was more than twice that of the group without pollinators (0.099 *vs* 0.042). The biggest difference in the ω ratios between the two groups was observed for the *Ans* gene, which demonstrated a nearly three-fold higher ratio in the group of plant species pollinated without attracting pollinators (0.196 *vs* 0.067). For the whole set of tested genes, the models dividing plant species into two groups based on pollination type resulted in significantly higher likelihoods than the single ratio model (Table 1). With exclusion of the *Chi* gene from the analysis, a strong positive correlation was found between dN/dS ratios of the structural genes and the positions of the enzymes encoded by the genes in the metabolic pathway in the group of plant species pollinated without pollinators (*r*_*s*_ = 0.97, *p* < 0.05) (Table 2).

Testing the hypothesis that regulation mode affects selective pressures acting on the genes revealed higher dN/dS ratios for the *F3h* gene in plant species in which the gene was regulated by ABP-specific regulatory factors and associated with color development (0.032 *vs* 0.127), whereas for the *Dfr* gene, the opposite tendency was observed with a slightly increased dN/dS ratio in plant species in which the gene was regulated independently of color development (0.098 *vs* 0.107) (Table 1, Fig. 3c). For the *F3h* and *Dfr* genes, a significant improvement in the likelihood was observed under the two-ratio model compared to the single ratio model. Although the dN/dS ratios for *Chs*, *Chi* and *Ans* were increased and decreased, respectively, in plant species in which the genes were regulated in a color-dependent fashion (0.051 *vs* 0.035 for *Chs*, 0.136 *vs* 0.119 for *Chi*, and 0.087 *vs* 0.106 for *Ans*), the single ratio model could not be rejected for these genes (Table 1).

## Discussion

### The ABP genes experienced purifying selection

Our data support previous findings that the core genes of the ABP are highly conserved and experienced purifying selection, likely due to their wide range of functions in plant physiology and the upstream genes being more constrained than the downstream genes [6–8]. In the current study, a comparison of the selective pressures between the genes under a single ratio model demonstrated that the genes can be divided into four groups: (I) *Chs*, (II) *Chi*, (III) *F3h* and (IV) a set of genes that contains *F3’h*, *Dfr* and *Ans*. These groups experienced significantly different selective pressures, with the strongest selective pressure acting on *Chs* and the weakest acting on *Chi*; a significantly indistinguishable selective pressure on the gene set was weaker than the selection acting on *F3h* (Additional file 4: Table S2). As has been demonstrated previously for structural and regulatory genes of different metabolic pathways, stronger selective pressures are experienced by genes with pleiotropic functions [5, 9, 12]. In the current study, except for *Chi*, the selective pressure acting on the ABP genes met this rule; *Chs* is the entry point in flavonoid metabolism, and all classes of flavonoids are synthesized with the participation of this enzyme, while *F3h* participates in the biosynthesis of a fewer number of flavonoid compounds. An indistinguishable selective pressure acted on the downstream set of genes *F3’h*, *Dfr* and *Ans,* suggesting a similar functional load and co-evolution of these genes. The upstream gene *Chi,* acting after *Chs,* does not meet the rule of minimum evolution. One of the probable explanations for the relaxed selective pressure on *Chi* is the possibility of spontaneous conversion of chalcones to flavanones without the participation of CHI [29].

There was no correlation between the genes and the positions in the pathway under the single ratio model, even when the selective pressure acting on the *Chi* gene was not included (Table 2). However, when two- or three-ratio models allowing different dN/dS ratios for distinct groups of the plant species were applied to the dataset, a correlation between the genes and positions in the pathway was detected in the group of the monocotyledonous plant species and in the group of plant species that were self- and wind-pollinated (Table 2). This observation allows us to suggest that distinct patterns of the ABP gene evolution in different groups of plant species occur.

### The factors affecting the selective pressures on the ABP genes

Comparing the likelihood functions generated under single and local ratio evolutionary models revealed that the ABP genes experienced different selective pressures in different lineages (Table 1). To test which factors could affect the selective pressures, we compared the single ratio model with the two- or three-ratio models, allowing separate ω for groups of the branches that were combined based on the class of the plant species (Fig. 2), pollination type (Fig. 2), and regulation mode (Fig. 1).

Comparing the improvement in the likelihood function constructed under the three hypotheses showed that pollination type has an association with selective pressure on the whole set of genes, with a greater selective constraint acting on the genes in the pollinator-dependent group of plant species, except for *Chs*, which was more constrained in the group of plant species pollinated without pollinators (Fig. 3b). Reportedly, the upstream genes can undergo duplications and divide broad functional pleiotropy among duplicated copies, which could relax the constraint on some of the gene copies [30, 31]. The observed relaxed constraint on the key flavonoid biosynthesis gene *Chs* in the pollinator-dependent group of plant species could be explained by a less functional load on the gene that probably occurred because the gene specialized in the biosynthesis of a reduced number of flavonoids classes. The duplication of the *Chs* gene has been reported in a broad diversity of plant species, including those chosen for this analysis [32, 33].

It is interesting that the *Ans* gene specific to anthocyanin biosynthesis is constrained almost three-fold in the pollinator-dependent group of species compared to the *Ans* from the pollinator-independent group. This observed pattern of molecular evolution with higher selective pressures acting on the ABP genes in the pollinator-dependent group of the plant species may be explained by the fact that the color of flowers is one of the features that attracts pollinators. It is one of the traits of pollination syndromes [34], and therefore, the genes responsible for color are additionally constrained compared to the genes from plant species in which the color has no such function (in self- or wind-pollinated plant species).

In dicots, the selective pressures on *F3h* and further downstream genes were stronger than in monocots, whereas the selective pressures on *Chs* and *Chi* were indistinguishable in plants belonging to different classes (Table 1, Fig. 3a). Most likely, the observed patterns for the evolution of *F3h* and more downstream genes could be explained by the pollination type because in the monocot group investigated, only one plant species, *A. andraeanum*, was pollinated by attracting pollinators (Fig. 2).

The regulation mode had some effects on the selective pressures acting on the ABP genes, and the most pronounced effect was on the *F3h* gene, in which there was an unexpectedly higher selective pressure on the *F3h* gene in plant species where the gene’s regulation was not dependent on ABP-specific regulatory factors or color development (Table 1). Using the principle of minimum pleiotropy, which implies that the genes with broader functionality are more constrained, we can hypothesize that the ‘not co-regulated’ *F3h* gene could be involved in the synthesis of diverse classes of flavonoids, including anthocyanins, whereas the functions of the ‘co-regulated’ *F3h* genes could be more associated with anthocyanin biosynthesis and less related to the synthesis of unpigmented flavonoids.

Such functional diversification could also occur after duplication events that could relax the constraint on all or some of the gene copies [30, 35]. In plant species chosen for the analysis, duplication of the *F3h* gene has been reported for wheat and related species [36]. One copy of *F3h* was specifically regulated by ABP-specific regulatory factors in pigmented tissues and expressed in other unpigmented organs, where its function is likely to be related to the biosynthesis of unpigmented classes of flavonoids, whereas the paralogous copy *F3h-B2* was not associated with pigment development and was specifically expressed in unpigmented roots only [36].

## Conclusions

Although the whole set of anthocyanin biosynthesis pathway genes is under purifying selection, suggesting its important functionality in plant life, the variation in selective pressures acting on the genes was revealed in different groups of plant species combined based on whether they belonged to the monocots or dicots, their pollination type, or regulation modes of the genes. Among the tested factors, only the pollination type had an impact on the selective constraints on the whole set of ABP genes, suggesting an important role of the pollination type in the evolution of anthocyanin biosynthesis, whereas the regulation mode had effects on the evolution of some of the pathway genes. The evolution of some of the genes such as *Chs* in the group of the pollinator-dependent plant species and such as *F3h* in the group of plant species where the gene is not strongly associated with pigmentation, could not be explained in the framework of the factors tested. We hypothesized effects of additional factors, such as multiple duplication and diversification of functions between gene copies, which could potentially explain the observed relaxed constraint on the *Chs* and *F3h* genes in some groups. The latter hypothesis should be tested by an appropriate dataset that combines data on duplicated gene sequences and their functions in the flavonoid biosynthesis pathway.

## Methods

### Dataset construction

For the analysis of the molecular evolution of the ABP genes, plant species covering broad taxonomic distances with the most characterized pathways regarding structural genes (*Chs*, *Chi*, *F3h*, *F3’h*, *Dfr*, *Ans*) and their regulatory patterns were chosen as follows: monocotyledons, *Ae. tauschii*, *A. andraeanum*, *H. vulgare*, *O. sativa*, *T. aestivum*, *T. urartu*, *Z. mays*; dicotyledons, *A. lyrata*, *A. thaliana*, *M. domestica*, *S. tuberosum*, *V. amurensis*, *V. vinifera*.

The known gene sequences of *A. thaliana*, *V. vinifera*, or *Z. mays* were used as query sequences for a BLAST search [37] of orthologous genes in the NCBI (www.ncbi.nlm.nih.gov/Database/), URGI (https://urgi.versailles.inra.fr/blast/blast.php), or BARLEX (http://apex.ipk-gatersleben.de/apex/f?p=284:10) (Additional file 2: Table S1) databases. The *Ans* gene of *T. urartu* was combined from two contigs retrieved from the URGI database (1,371,254 and 1,363,707). The missing part of the gene was sequenced by applying the primer pair, Forward: 5’ATGCCGATCGAGGACAA3’, and Reverse: 5’TTGGTGAGGATGAAGGAGAG3’, designed using the PrimerQuest Tool software (https://eu.idtdna.com/Primerquest/Home/Index) (Additional file 5).

To ensure the quality (as well as to exclude putative rare intra-species SNPs or sequencing mistakes) of the retrieved and annotated sequences, they were blasted to NCBI database and compared with homologous sequences. All sequences further used in our comparative studies passed this quality test successfully.

The nucleotide sequences of the genes were converted to their corresponding peptide sequences and aligned by MULTALIN 5.4.1 [38]. The amino acid sequence alignments were manually edited to eliminate signal sequences at the N- and C-termini of the peptides, as well as indels and gaps. Subsequent conversion of the edited peptide alignments to corresponding nucleotide sequence alignments allowed maintenance of the coding reading frame for further codon-based analysis.

A phylogenetic tree was constructed from the combined ABP gene sequences based on the bootstrap neighbor-joining (NJ) method with a Kimura 2-parameter model by MEGA v5.1 [39]. The stability of internal nodes was assessed using bootstrap analysis with 1000 replicates.

The regulation patterns of the genes in the species chosen were taken from the published articles and schematically combined together in Fig. 1.

### Molecular evolutionary analyses

Codon-based maximum likelihood (ML) methods incorporated in the HyPhy package [40] were applied to determine the ratios of non-synonymous to synonymous substitutions (dN/dS or ω) which provide a measure of the selective pressure at the protein level, where ω < 1 suggests purifying selection, and ω = 1 and ω > 1 indicate neutral evolution and positive selection, respectively. A general time-reversible model (GTR or REV) was applied as a nucleotide substitution model [41]. The ω value for each of the genes was assessed under the global (or one-ratio) model that has a shared ω for all branches. The ω values were compared between the genes using the method described by Ho and Smith [15]. For this analysis, the genes were grouped into all possible pairs. The likelihood of the model allowing for different codon frequencies and substitution rates for each gene but fitting a single dN/dS ratio (ω) was compared with the likelihood of the model estimating a separate ω for each of the genes by applying the likelihood ratio test [42].

Spearman’s rank correlation coefficients were calculated between the ω ratio of the genes and the positions of the enzymes encoded by the genes in the metabolic pathway.

To test the influence of different factors on the selective pressures acting on the ABP genes, the global model was compared with the branch models that had separate ω values estimated for groups of branches combined to test various hypotheses. For significance testing, the likelihood ratio test was applied.

The effects of three factors were tested using the constructed dataset: class of the plant species (two separate ω for each of the ABP genes were calculated for monocots and dicots, ω_m_/ω_d_); pollination type (the ω for the plant species pollinated by pollinators was calculated separately from the other plant species pollinated without the attraction of pollinators, ω_p_/ω_np_); regulation pattern (two separate ω for each of the ABP genes were calculated for the plant species in which the gene was strongly regulated by the ABP regulatory factors and associated with pigmentation and for plant species in which the gene was regulated independent of color development; the ω ratios were called color-dependent and color-independent, ω_cd/_ω_ind_). The combining of the plant species and internal nodes based on the hypothesis-testing are presented in (Additional file 6: Figure S3). To combine the plant species chosen for molecular analysis based on their regulation mode, two assumptions were made: (1) the closely related species had similar patterns of the ABP gene regulation (e.g.*,* a known pattern of regulation for *A. thaliana* was shared with *A. lyrata*, where the regulation pattern had not been known); (2) the internal nodes leading to lineages with color-dependent as well as with color-independent regulation patterns were combined with species in which the genes were regulated independently of anthocyanin biosynthesis.

## Additional files

Additional file 1: Figure S1. Schematic representation of the flavonoid biosynthesis pathway in plants according to Khlestkina et al. (2015). (DOCX 244 kb)

Additional file 2: Table S1. Accession numbers of the gene sequences used in the current study identified in the NCBI, URGI or BARLEX databases. (DOCX 25 kb)

Additional file 3: Figure S2. Gene trees for *Chs*, *Chi*, *F3h*, *F3’h*, *Dfr*, and *Ans*. (DOCX 209 kb)

Additional file 4: Table S2. dN/dS comparisons across genes. (DOCX 18 kb)

Additional file 5: The reconstructed full-length nucleotide sequence of the *Ans* gene in *T. urartu*. (DOCX 13 kb)

Additional file 6: Figure S3. The phylogenetic tree of the plant species selected for testing the effects of plant classes (A), pollination type (B) and regulation mode (C1-C5) on selective pressures acting on the structural genes. (DOCX 159 kb)

## Abbreviations

ABP: Anthocyanin biosynthesis pathway
ANS: Anthocyanidin synthase
AT: Acetyltransferase
CHI: Chalcone-flavanone isomerase
CHS: Chalcone synthase
DFR: Dihydroflavonol 4-reductase
F3’H: Flavonoid 3′-hydroxylase
F3H: Flavanone 3-hydroxylase
GT: Glycosyltransferase
MT: Methyltransferase
RT: Rhamnosyltransferase
