# Supplementary material for: The factors affecting the evolution of the anthocyanin biosynthesis pathway genes in monocot and dicot plant species
Source: BMC Plant Biol. 2017 Dec 28;17(Suppl 2):256. doi: 10.1186/s12870-017-1190-4 (PMC5751542; doi:10.1186/s12870-017-1190-4)
Supplement: Supplementary file 1 — Schematic representation of the flavonoid biosynthesis pathway in plants according to Khlestkina et al. (2015). (DOCX 244 kb) [file 12870_2017_1190_MOESM1_ESM.docx]

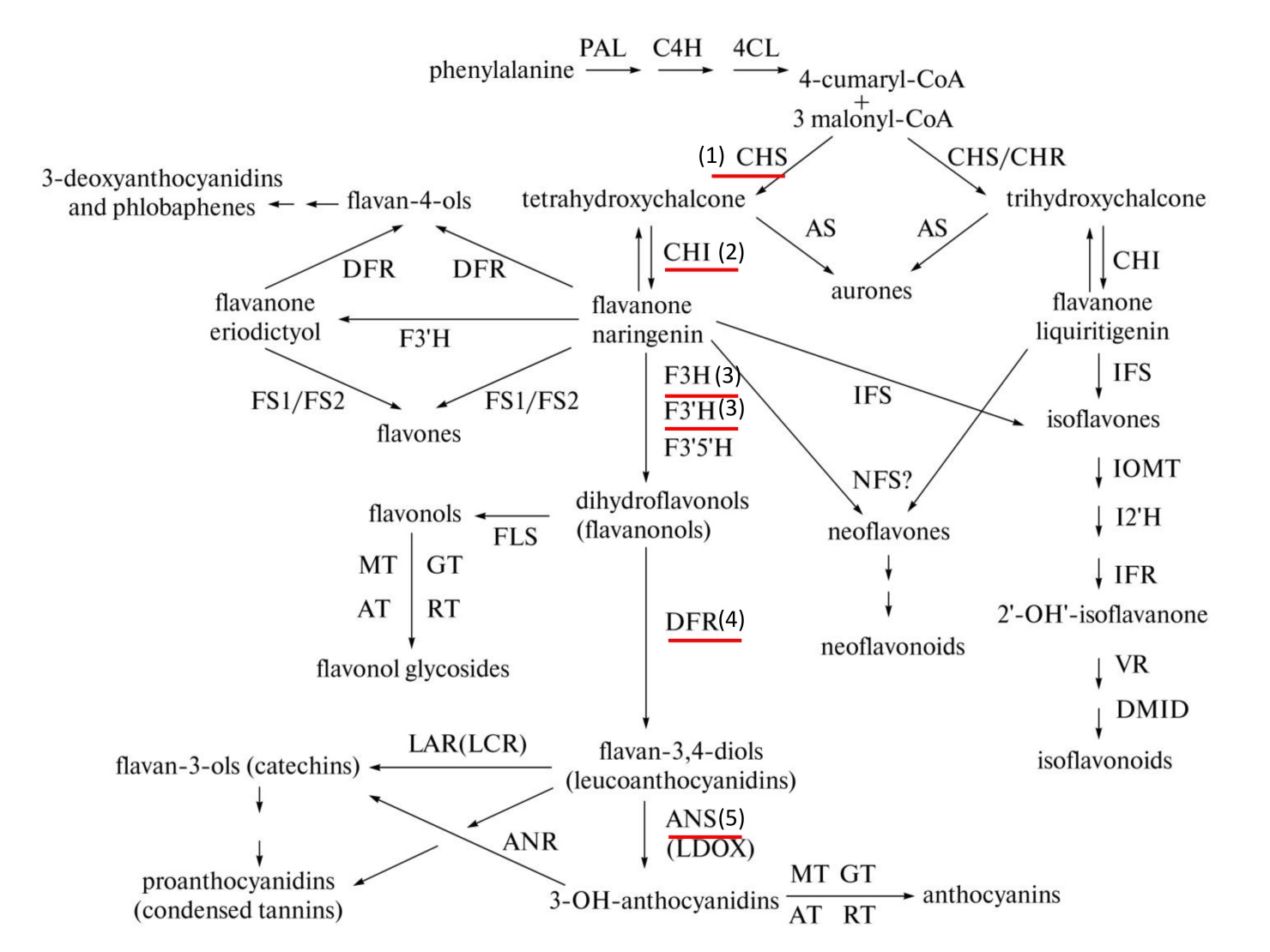


**Figure S1.** Schematic representation of the flavonoid biosynthesis pathway in plants according to Khlestkina et al. (2015). 4CL – 4-Cumarat: CoA-Ligase; ANR – anthocyanidin reductase; ANS (LDOX) – anthocyanidin synthase (leucoanthocyanidin dioxygenase); AS (AUS) – aureusidin synthase; AT – acetyltransferase; CHI – chalconeflavanone isomerase; CHR – chalcone reductase; C4H – cinnamat-4-hydroxylase; CHS – chalcone synthase; DFR – dihydroflavonol 4-reductase; DMID – 7,2'-dihydroxy-4'-methoxyisoflavanol dehydratase; F3H – flavanone 3-hydroxylase; F3'H – flavonoid 3'-hydroxylase; F3'5'H – flavonoid 3',5'-hydroxylase; FLS – flavonol synthase; FS (FNS) – flavone synthase; GT – glycosyltransferase; I2'H – isoflavone 2'-hydroxylase; IFR – isoflavone reductase; IFS – isoflavone synthase; IOMT – isoflavone *O*-methyltransferase; LAR (LCR) – leucoanthocyanidin reductase; МТ – methyltransferase; NFS – neoflavone synthase; PAL – phenylalanine ammonia-lyase; RT – ramnosyltransferase; VR – vestitone reductase.

The enzymes leading to the anthocyanin pigments and its position number in the pathway branch are underlined.
