# Supplementary material for: The factors affecting the evolution of the anthocyanin biosynthesis pathway genes in monocot and dicot plant species
Source: BMC Plant Biol. 2017 Dec 28;17(Suppl 2):256. doi: 10.1186/s12870-017-1190-4 (PMC5751542; doi:10.1186/s12870-017-1190-4)
Supplement: Supplementary file 2 — Accession numbers of the gene sequences used in the current study identified in the NCBI, URGI or BARLEX databases. (DOCX 25 kb) [file 12870_2017_1190_MOESM2_ESM.docx]

**Table S1.** Accession numbers of the genes sequences used in the current study identified in NCBI (https://www.ncbi.nlm.nih.gov/), URGI (<https://wheat-urgi.versailles.inra.fr/>), or BARLEX (<http://apex.ipk-gatersleben.de/apex/f?p=284:10>) databases.

|  | **Accession number,**  **coding sequence length (bp) / length of the sequence used in molecular evolution analysis (bp)** | | | | | |
| --- | --- | --- | --- | --- | --- | --- |
| **Species** | ***Chs*** | ***Chi*** | ***F3h*** | ***F3’h*** | ***Dfr*** | ***Ans*** |
| *Arabidopsis thaliana* | AF112086, 1189/1146 | BT005528, 741/591 | NM_114983, 1077/939 | NM_120881, 1542/1278 | NM_123645, 1149/921 | NM_001036623, 1071/972 |
| *Arabidopsis lyrata* | AF112100, 1191/1146 | XM_021026334, 735/591 | XM_002876029, 1077/939 | XM_002871252, 1545/1278 | XM_002863688, 1155/921 | XM_021017925, 1071/972 |
| *Vitis vinifera* | NM_001280950, 1182/1146 | NM_001281104, 705/591 | NM_001281105, 1092/939 | NM_001280987, 1530/1278 | AY780886, 1014/921 | NM_001281218, 1068/972 |
| *Vitis amurensis* | KT589834, 1182/1146 | KT314072, 705/591 | KP966099, 1092/939 | FJ645766, 1530/1278 | FJ645768, 1014/921 | FJ645769, 1068/972 |
| *Solanum tuberosum* | KF285826, 1170/1146 | XR_367385, 741/591 | HQ659496, 1077/939 | XM_006345070, 1545/1278 | HQ659495, 1149/921 | HQ701728, 1368/972 |
| *Malus domestica* | DQ026297, 1170/1146 | XM_008388244, 687/591 | AF117270, 1095/939 | FJ919631, 1536/1278 | NM_001293939, 1047/921 | AF117269, 1074/972 |
| *Anthurium andreanum* | DQ421809, 1176/1146 | KU356776, 777/591 | AY232493, 1113/939 | KJ624416, 1536/1278 | DQ364060, 1044/921 | EF079869, 1092/972 |
| *Zea mays* | AY728478, 1203/1146 | Z22760, 696/591 | NM_001136803, 1101/939 | HQ699781, 1554/1278 | NM_001158995, 1074/921 | NM_001112604, 1188/972 |
| *Oryza sativa* | AB058397, 1197/1146 | AF474922, 702/591 | NM_001060692, 1167/939 | HQ876708, 1581/1278 | AB003495, 1119/921 | NM_001049600, 1128/972 |
| *Hordeum vulgare* | X58339, 1197/1146 | AF474923, 696/591 | X58138, 1134/939 | AK362052,  1557(partial)/1278 | S69616, 1065/921 | 941389, 1630795, 1993730^7^ (Shoeva et al. 2016), 1197/972 |
| *Triticum aestivum* (A) | 5222811^1^, 1185/1146 | JN039037, 690/591 | EF463100, 1137/939 | 4383986^4^, 1584/1278 | AB162139, 1065/921 | AB247918, 1182/972 |
| *Triticum aestivum* (D) | 5335048^2^, 1185/1146 | JN039039, 696/591 | DQ233636, 1137/939 | 2099349^5^, 1563/1278 | AB162140, 1065/921 | AB247921, 1317/972 |
| *Triticum urartu* | 189017^3^, 1170/1146 | KF826812, 690/591 | 180441^3^, 1134/939 | 186175^3^, 1566/1278 | AB276086, 1065/921 | 1371254, 1363707^3^*, 1182/972 |
| *Aegilops tauschii* | XM_020311362, 1185/1146 | KF826813, 696/591 | DQ233637,  1089(partial)/939 | 95596^6^, 1563/1278 | AB276101, 1065/921 | XM_020315529, 1317/972 |
| *Ginkgo biloba* | AY496931, 1176/1146 | Cheng et al. 2011, 735/591 | AY742228, 1074/939 | KP056747, 1671/1278 | KC847053, 1035/921 | EU600206, 1065/972 |

^1^ the contig was identified in URGI ‘Wheat sequence survey V2 chromosome 2AS’ database; ^2^ the contig was identified in URGI ‘Wheat sequence survey V2 chromosome 2DS’ database; ^3^ the contig was identified in URGI ‘Urartu v1’ database; ^4^ the contig was identified in URGI ‘Wheat sequence survey V2 chromosome 6AS’ database; ^5^ the contig was identified in URGI ‘Wheat sequence survey V2 chromosome 6DS’ database; ^6^ the contig was identified in URGI ‘Tauschii v1’ database; ^7^ the contigs were identified in BARLEX ‘assembly_WGSBowman’ database; * full nucleotide sequence of the gene was determined in the current study (File S1).

**References**

Cheng H., Li L., Cheng S., Cao F.,Wang Y., Yuan H. 2011. Molecular cloning and function assay of a chalcone isomerase gene (GbCHI) from *Ginkgo biloba*. Plant Cell Rep. 30, 49–62.

Shoeva O.Yu., Mock H-P, Kukoeva T.V., Börner A., Khlestkina E.K. 2016. Regulation of the flavonoid biosynthesis pathway genes in purple and black grains of *Hordeum vulgare*. PLoS ONE 11(10): e0163782. doi:10.1371/journal.pone.0163782
