# Supplementary material for: The factors affecting the evolution of the anthocyanin biosynthesis pathway genes in monocot and dicot plant species
Source: BMC Plant Biol. 2017 Dec 28;17(Suppl 2):256. doi: 10.1186/s12870-017-1190-4 (PMC5751542; doi:10.1186/s12870-017-1190-4)
Supplement: Supplementary file 3 — Gene trees for Chs, Chi, F3h, F3’h, Dfr, and Ans. (DOCX 209 kb) [file 12870_2017_1190_MOESM3_ESM.docx]

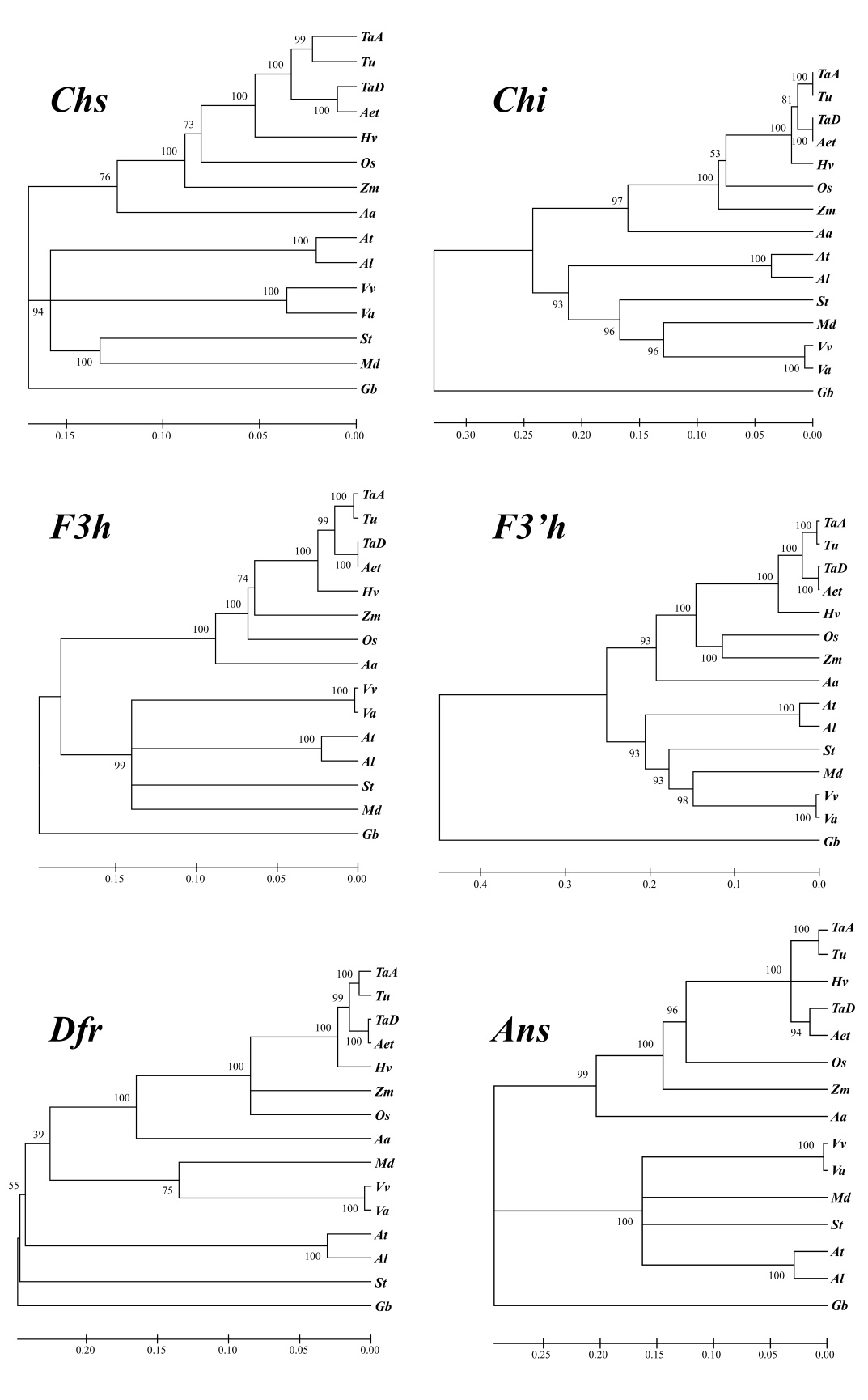


**Figure S2.** Gene trees for *Chs*, *Chi*, *F3h*, *F3’h*, *Dfr*, and *Ans*. The scale is in nucleotide substitutions per site, estimated by neighbor-joining (for genes except for *Chi*) and UPGMA (for *Chi* only) algorithms under a Kimura 2-parameter model using MEGA 6.06. The species names are abbreviated as follows: *Aa* – *Anthurium andreanum*, *Aet* – *Aegilops tauschii*, *Al* – *Arabidopsis lyrata*, *At* – *Arabidopsis thaliana*, *Gb* – *Ginkgo biloba*, *Hv* – *Hordeum vulgare*, *Md* – *Malus domestica*, *Os* – *Oryza sativa*, *St* – *Solanum tuberosum*, *TaA* – *Triticum aestivum* (A), *TaD* – *Triticum aestivum* (D), *Tu* – *Triticum urartu*, *Va* – *Vitis amurensis*, *Vv* – *Vitis vinifera*, *Zm* – *Zea mays*.
