# Supplementary material for: The factors affecting the evolution of the anthocyanin biosynthesis pathway genes in monocot and dicot plant species
Source: BMC Plant Biol. 2017 Dec 28;17(Suppl 2):256. doi: 10.1186/s12870-017-1190-4 (PMC5751542; doi:10.1186/s12870-017-1190-4)
Supplement: Supplementary file 4 — dN/dS comparisons across genes. (DOCX 18 kb) [file 12870_2017_1190_MOESM4_ESM.docx]

**Table S2.** dN/dS comparisons across genes. Model C allows for different codon frequencies and substitution rates for each genes, but fits a single dN/dS ratio (ω). Model E estimates a separate ω for each partition (listed in Table in the order of the genes listed). * Significant increase in likelihood (*p*<0.05).** Significant increase in likelihood (*p*<0.001).

| **Partition** | **Model** | **Ln *L*** | **ω** |
| --- | --- | --- | --- |
| All genes  (*Chs*, *Chi*, *F3h*, *F3’h*, *Dfr*, *Ans*) | C | -42402.41 | 0.074 |
|  | E | -42331.12** | 0.036, 0.121, 0.061, 0.085, 0.095, 0.094 |
| *Chs*, *Chi* | C | -11999.37 | 0.056 |
|  | E | -11953.74** | 0.036, 0.121 |
| *Chs*, *F3h* | C | -13345.67 | 0.046 |
|  | E | -13335.96** | 0.036, 0.061 |
| *Chs*, *F3’h* | C | -17886.15 | 0.056 |
|  | E | -17855.04** | 0.036, 0.085 |
| *Chs*, *Dfr* | C | -14350.36 | 0.056 |
|  | E | -14314.50** | 0.036, 0.095 |
| *Chs*, *Ans* | C | -15250.56 | 0.056 |
|  | E | -15215.20** | 0.036, 0.094 |
| *Chi*, *F3h* | C | -10133.60 | 0.081 |
|  | E | -10118.03** | 0.121, 0.061 |
| *Chi*, *F3’h* | C | -14642.08 | 0.096 |
|  | E | -14637.13* | 0.121, 0.085 |
| *Chi*, *Dfr* | C | -11098.69 | 0.105 |
|  | E | -11096.58* | 0.121, 0.095 |
| *Chi*, *Ans* | C | -11999.59 | 0.104 |
|  | E | -11997.27* | 0.121, 0.094 |
| *F3h*, *F3’h* | C | -16024.16 | 0.074 |
|  | E | -16019.34* | 0.061, 0.085 |
| *F3h*, *Dfr* | C | -12486.61 | 0.076 |
|  | E | -12478.81** | 0.061, 0.095 |
| *F3h*, *Ans* | C | -13387.02 | 0.076 |
|  | E | -13379.50** | 0.061, 0.094 |
| *F3’h*, *Dfr* | C | -16998.50 | 0.089 |
|  | E | -16997.88 | 0.085, 0.095 |
| *F3’h*, *Ans* | C | -17899.10 | 0.089 |
|  | E | -17898.59 | 0.085, 0.094 |
| *Dfr*, *Ans* | C | -14358.05 | 0.095 |
|  | E | -14358.04 | 0.095, 0.094 |
