# Supplementary material for: The factors affecting the evolution of the anthocyanin biosynthesis pathway genes in monocot and dicot plant species
Source: BMC Plant Biol. 2017 Dec 28;17(Suppl 2):256. doi: 10.1186/s12870-017-1190-4 (PMC5751542; doi:10.1186/s12870-017-1190-4)
Supplement: Supplementary file 5 — The reconstructed full-length nucleotide sequence of the Ans gene in T. urartu. (DOCX 13 kb) [file 12870_2017_1190_MOESM5_ESM.docx]

The reconstructed full-length nucleotide sequence of the *T. urartu* *Ans* gene. Yellow marks the coding sequence. Primer pair designed for sequencing is underlined.

**>T_urartu_ANS**

cgaggaggaggattgatgtgtctttcgtttgtgtcttgaacttggtttgcattttgaacttggttggataaacttgtgggcatgaacttttatttatcaatttgtttgtgtgaaaatttatatgtcatgttcattacattttgaatgttttcaaaattcattttgtgtccaaaatgccatatatgcaatgcctcggcgagtcacgtgcgctgcattttttgcgcactgctggagcggcgtgcgttgcattttagcgcggctgttggagccagcgctggcggccgcgcaaaaccagacgaacggcgcgcagcaaactagtttttagcgcgcggcgcgtaccgcggctgttggagatggtctgagcttagggttctttggttgctactatttttcttttttgctccttgtctagggaaggaagaaaacagttatctcttttgaagggaagcaaaaaaaaaaagtgcaggttgaaacatgaggctagggcctacatgaggtgcagcccagcgtgacagcatctcgtgtcggcatcgatatcacagcacgcactgtttctgctaaggagattacagtttggtagcacaaaaaaaacaaaaaatggagttggacgatagcatgcagcatgtgcgtgcgtgtggttggAAGCGAAATCTGCTCTGCTTTTGGCGCGTGATGGAAAGTTGGAGCTAACCACCTAACCAAAACCAAGCCATGGATTCTTCTCATCTCTCATCTACTATAAGTAAACCATCCATGAACTCCATCGATTCTCGTCGCAACTAATCCGATCACCAAGTGATAGCATACCTCCGGAGGAGGAGGAAGAAGAAGAATCAAACTGCGATGGCGCGGGTGGAGGCGCTGAGCATGAGCGGCGCGACGACGATCCCGGCGGAGTACGTGCGGCCGCAGGAGGAGCGCCAGGGCCTGGGCGACGCCTACGCCGAGGCGGCGGCCTGCTGGTCCGCGGCGGGCTCCCCTCGAATCCCCATCGTCGACGTGGCCGCCTTCGACGCCGCGGACCCGGCCTCCCCAGCGAGCCTTGCCGTCGTTGACGCCGTGCGCGCCGCCGCGGAGGACTGGGGCGTCATGCACCTGGCCGGTCACGGCATCCCGGAGGATCTCATCGATGCACTGCGCGGCGCCGGCACGGGGTTCTTCCGCATGCCGATCGAGGACAAGGAGGCCTACGCCACGACCCCGGCGGCGGGGAGGTTGGAGGGGTACGGCAGCCGGCTCGCCGGATCCGCCGGCGAGGACGGGAAGAGGGAGTGGGAGGACTTCCTGTTGCACATGCTCCACCCCGACGCCCGCGCCGACCACGCGCTCTGGCCGGCATACCCGCCCGAGTACGTGCCCGTCACCAAGAGCTTCGGGGAGCACGTGAGCGCGCTCTCGTCCCGGCTCCTCGCCATCCTCTCCCTCGGGCTCGGCGTCCAGGCCGGCACCCTCGAGCGCCGCCTCCGCCTCACCTCCGGCGAGGCCCAGGTGGAGGACGACCTGCTGCTGAAGCTCAAGATCAACTACTACCCGCGGTGCCCGCAGCCGGAGCTGGCCGTGGGCGTGGAGGCGCACACCGACGTGTCCGCGCTCTCCTTCATCCTCACCAAAGGCGTGCCGGGCCTGCAGGTCCTCCGTCCCGGCGACGGCCAGACCTGGGTCACCGCGCGTGACGAGCCGGGCACGCTCGTGGTCCACGTCGGCGACGCGCTGGAGATCCTCAGCAACGGGCGCTACACCAGCGTGCTCCACCGCGTGCTCGTCAACCGGCAGGCCGTGCGAGTCTCCTGGGTCGTCTTCGCCCAGCCGCCGCCCGACTCCGTGCTGCTGCGCCCGATGCCGGAGCTCGTCCAAGGGGATGGCGCCGAGACGCCGCGCTTCGAGCCGCGCACCTTCAGGCAGCAGCTCGAGCGCAAGGTCCTCAAGAAGACGAATGATCAACAAGAGGACTAGGTTAAGAAGCAGCCGGTCGCCGCCGGTCGTCAGCGAGGAGGAGCACATGGTCGTGAAGAAGGAGCAGAGTGAACCAGAAGAGGAGGCCAAGATTGGAGAGGCACCGCTGACAGCTAACCTAGTAGTAGAAGTTAACTAACTAAATAATGAATTGATCCAGTTTCTCTCAGAATTGCCATTTCATTTTTTTTACAATTCCTAGCTAAATAATGTTTAGTTAGGTATGAATCGTAAAAAAATTGAAGTGACAATTCTGGAAAAATTGCCAAAAAAACTGGTCGTATATGGAACGCAAAGCAAAGCCAAACCACAATACACAGCTTCTTCGCCAAAACATAAATACAAATCATTTCGGCAATGCGAAAACCCAAAGGTGGCATCATCAGCCAGCCAACACAGTCCTGTCTTCATCGGAAACCACCCTTTGCCTTCGACACATCATCATCTTCTTCGCTGCCTTCGGGTTGGCCTGCACCAAACCCACACACGTGTTTATTTTTGTTTGGCGCATCTCGGCTTCCCACGGATCCATTTCTCTTTTGAGCACATCTCGGCTTCCCACAAATAACAAGTTTCACATTTTTTTTAAATAACAAGTTCCGCCGCCCGTTGCCATGGGCTCACACTCGTGAGCGTCCGAAGCCGAAGGGAAGAAGTGGCCCCGCCGCCACCGGATCCCGGCGGGCTTCTCCCGGCGGAGTCCACCAGTGACGTCGAGGGGAGAGGAGGGGGCGTGGTGGCTAGGAGGCAGACGCGAGGGCTGGTTAGTTTTTGCGGACAGTTTTACACGAGACAAGTACTCCCTCTGTTCGGTGAAAAGTGTACATTTAACTTTAAAATTTGTTCATAAAAGAATGTACTTCTATCCTCTCAATGCACGTAAAGTAGGAAAAAAATGATTCTCCCTCATCACACGGTAATCAAGATCAATAACAATTACATATGGTTTCCTTAATTTCTACACAAACTTGGCTCCTTGGAGATTGGATAATTAAAGAGAAGAGAGATGATGGCTTGCACATTTTCAATGCATTTTTACCGCACTTCATAATTTATTGTAAAATTTCTACATGTACTCAATGTGAGTACACTTTCCACCGAACAGAAGAAGTAAAGATTGTAAGTATTGTAAGTTTGTAAGAAACCACATGCCAGTGACACAAGTAGGCTACTACTAGTAGCGGCCATGCATGCTACTGTACGTTCCTAAAAAAATAGTAGTACTGTTGCGTGCTTAGAAAGTGCATCTGGTAATTAATGCATGCACGACGGGCGAAATGCACAAAGAATAAAGAAAAAAAGAAAAGATCATGAGAGGAGTTGCGGCCTCTCTTGCTCACGTAAGGCTGCTGCCAATGCATGGGTGCTTAGATAAGGTGCTAAGCACATTAAATAGCTTAGCAACTATACTCCCCA
